# A regulatory loop between miR-132 and miR-125b involved in gonadotrope cells desensitization to GnRH

**DOI:** 10.1038/srep31563

**Published:** 2016-08-19

**Authors:** Jérôme Lannes, David L’hôte, Ambra Fernandez-Vega, Ghislaine Garrel, Jean-Noël Laverrière, J -C -T Joëlle-Cohen-Tannoudji, Bruno Quérat

**Affiliations:** 1 Université Paris-Diderot, Sorbonne Paris Cité, Biologie Fonctionnelle et Adaptative (BFA), F-75013 Paris, France; 2 Centre National pour la Recherche Scientifique (CNRS) UMR 8251, Paris, France; 3 Physiologie de l’axe gonadotrope, Institut National de la Santé et de la Recherche Médicale (INSERM) U1133 Paris, France

## Abstract

The GnRH neurohormone is the main activator of the pituitary gonadotropins, LH and FSH. Here we investigated the contribution of microRNAs in mediating GnRH activation. We first established that miR-125b targets several actors of Gαq/11 signalling pathway, without altering Gαs pathway. We then showed that a Gαs-mediated, PKA-dependent phosphorylation of NSun2 methyltransferase leads to miR-125b methylation and thereby induces its down-regulation. We demonstrated that NSun2 mRNA is a target of miR-132 and that NSun2 may be inactivated by the PP1α phosphatase. Time-course analysis of GnRH treatment revealed an initial NSun2-dependent down-regulation of miR-125b with consecutive up-regulation of LH and FSH expression. Increase of miR-132 and of the catalytic subunit of PP1α then contributed to NSun2 inactivation and to the return of miR-125b to its steady-state level. The Gαq/11-dependent pathway was thus again silenced, provoking the down-regulation of LH, FSH and miR-132. Overall, this study reveals that a regulatory loop that tends to maintain or restore high and low levels of miR-125b and miR-132, respectively, is responsible for gonadotrope cells desensitization to sustained GnRH. A dysregulation of this loop might be responsible for the inverted dynamics of these two miRNAs reported in several neuronal and non-neuronal pathologies.

The gonadotropin-releasing hormone (GnRH) is a decapeptide secreted by hypothalamic neurones into the pituitary portal system. Upon binding to its receptor (GnRHR) on pituitary gonadotrope cells, GnRH stimulates the synthesis and secretion of two gonadotropins, luteinizing hormone (LH) and follicle-stimulating hormone (FSH)^1^. Gonadotropins are heterodimers of two glycoproteins, a common α-subunit and a specific, rate-limiting β-subunit (LHβ and FSHβ). Secreted gonadotropins stimulate gonadal growth, steroidogenesis and gametogenesis. GnRHR is a 7-domain trans-membrane receptor^2^ that couples with both Gαq/11 and Gαs to activate phospholipase Cβ (PLCβ) and cAMP downstream signalling, respectively^3^,^4^,^5^. GnRHR presents a unique feature in that it lacks an intra-cytoplasmic C-terminal tail^2^. It is therefore not subject to homologous desensitization and rapid internalization^6^. However, sustained exposure to GnRH, after an initial transient activation, leads to repression of gonadotropin expression and secretion^7^, resulting in low levels of circulating gonadal steroids. The efficacy of this action has long been proved in treatment against endometriosis, central precocious puberty, polycystic ovary syndrome or cancers of gonadal steroid-dependent tissues, with low toxicity^8^,^9^. Desensitization to GnRH has been proposed to be mediated by a down-regulation of Gαq/11 signalling^10^,^11^,^12^. However, the mechanism of this down-regulation has not been precisely described yet and, notably, the role of microRNAs (miRNAs) in desensitization processes has still to be explored.

MicroRNAs (miRNAs) are small (21–24 nt) single stranded RNAs that regulate gene expression at a post-transcriptional level^13^. They act through base pairing to complementary regions of their target mRNAs within the RNA-induced silencing complex (RISC). This results in down-regulation of target expression at transcript and/or translational level^14^,^15^. The gonadotrope-specific deletion of dicer, an endoribonuclease involved in the biogenesis of miRNAs, completely abolished the synthesis of the two gonadotropins leading to male and female infertility^16^,^17^. GnRH treatment of murine immortalized LβT2 gonadotrope cells was shown to modulate expression of several miRNAs^18^,^19^. We recently demonstrated that the rise in two of the most induced-ones, miR-132 and miR-212 was necessary for the stimulation of FSH expression^20^. Conversely, miR-125b was found to be among the most repressed miRNAs^18^,^19^. This was particularly interesting as miR-125b and miR-132 have been shown to exhibit opposing effects on dendritic spine morphology and synaptic physiology in hippocampal neurons^21^. If such opposing effects were to occur in gonadotrope cells, the inverse behaviour following GnRH exposure, *i.e.* increased miR-132 and decreased miR-125b, should contribute to the activation of gonadotropins expression. Encouragingly, miR-125b was demonstrated on different cell models to target the mRNA of several cellular components like MAP2K7^22^,^23^, p38^23^ and JUN^24^, all three known to be involved in Gαq/11-mediated GnRH signalling^4^, The present study was aimed to address the role of miR-125b in the GnRH signalling with a particular attention on a possible contribution to the desensitization mechanism.

## Results

### miR-125b inhibits gonadotropins expression

**Figure 1:**
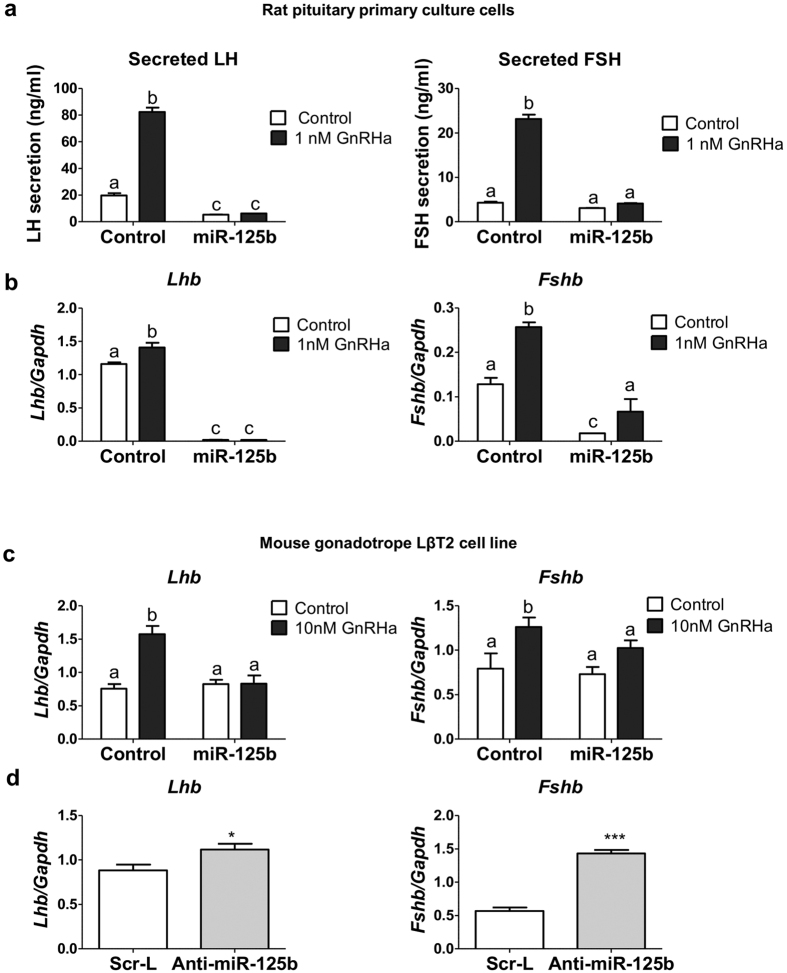
miR-125b is involved in the GnRH induction of LH and FSH expression. (**a,b**) Rat primary pituitary cells were electroporated with a miR-125b expressing vector or an empty vector and then treated with 1 nM GnRHa for 4 hours. (**a**) The concentration of accumulated LH and FSH into the medium was measured by ELISA. Overexpressing miR-125b significantly reduced basal secretion of LH and prevented the GnRHa-induced secretion of LH and FSH (n = 4). (**b**) *Lhb* and *Fshb* mRNA levels were measured by qRT-PCR and normalized to *Gapdh* mRNA. Overexpressing miR-125b nearly abolished basal expression of both *Lhb* and *Fshb* mRNA and prevented their GnRH-induced expression (n = 3). (**c**) LβT2 cells were electroporated with a miR-125b expressing vector or an empty vector and then treated with 10 nM GnRHa for 4 h. Overexpression of miR125 prevented the GnRHa-increased *Lhb* and *Fshb* mRNA expression (n = 7). (d) LβT2 cells were electroporated with anti-miR-125b or scrambled LNA. Blocking miR-125b increased both LHb and Fshb mRNA levels (n ≥ 10). Different letters illustrate significant differences. *P < 0.05; **P < 0.01; ***P < 0.001.

Overexpression of miR-125b in control or GnRHa-treated (1 nM for 4 h) rat pituitary cells significantly decreased basal LH secretion and prevented GnRH-induced secretion of both LH and FSH (Fig. 1a). *Lhb* and *Fshb* steady-state mRNA levels were strongly reduced when miR-125b was overexpressed in control as well as in GnRH-treated rat pituitary cells (Fig. 1b).

Gonadotrope cells represent less than 15% of pituitary cells. To decipher the mechanism of miR-125b action, we thus used the murine LβT2 gonadotrope cell model. Overexpression of miR-125b in LβT2 cells (Fig. S1a) had no effect on basal gonadotropin β subunits mRNA level (Fig. 1c), probably because of an already low (hundred times lower) expression level. However, as in rat pituitary cells, it abolished the GnRH-induced (10 nM for 4 h) increase observed in control cells (Fig. 1c). Conversely, blocking miR-125b action (Fig. S1a) induced an increase in *Lhb* and *Fshb* mRNA level (Fig. 1d). These effects occurred at a transcriptional level as either overexpression or blocking miR-125b significantly altered both *Fshb* and *Lhb* promoter activities in LβT2 cells (Fig. S1b).

### miR-125b prevents GnRH activation of Gαq/11-, but not Gαs-dependent pathway

A number of potential targets for miR-125b that were predicted from *in silico* analyses are known to be involved in GnRH signalling^25^. Some of these potential targets (*Map2k7, Jun* and *Mapk14* (encoding p38), all depending on PLCβ activation) were confirmed in different cell types^22^,^23^,^24^. In addition to these targets, we investigated the potential effect of miR-125b on Gαq/11, to which GnRH receptor couples, CACNA1C that controls calcium entry, ELK1, IP3-R (encoded by *Itpr1*) and CAMK2a that are responsible for and activated by calcium release, respectively (see Fig. S1c for a schematic presentation of GnRH signalling pathways).

**Figure 2:**
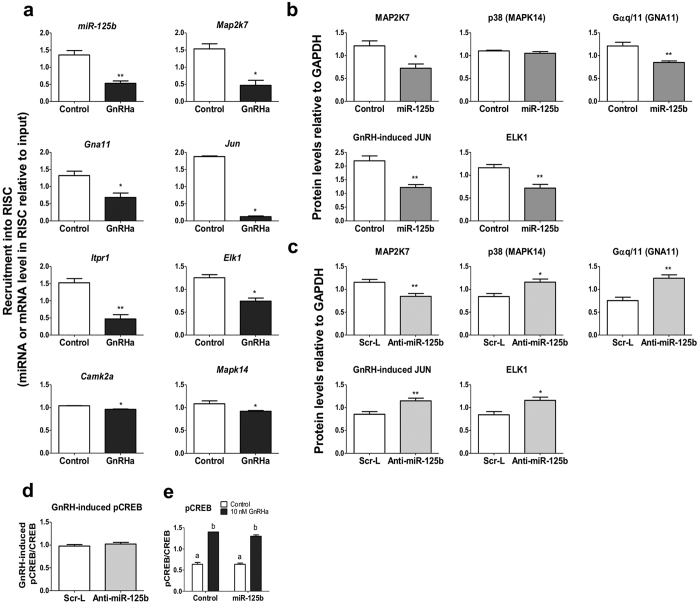
miR-125b prevents GnRH activation of Gαq/11-, but not Gαs-dependent pathways. (**a**) Effect of a GnRHa treatment on the recruitment into RISC of Gαq/11-medated pathway factors. Levels of miR-125b and selected potential target mRNAs were determined by qRT-PCR on pan-AGO-immunoprecipitated samples and normalized to input level. GnRHa treatment (10 nM for 4 h) of LβT2 cells reduced the recruitment of miR-125b and of all tested target mRNAs (n = 5). (**b,c**) Effect of alteration of miR-125b on the protein level of Gαq/11-related pathway factors in LβT2 cells. b; Cells were electroporated with a miR-125b expressing vector or an empty vector and protein levels were quantified on harvested cells by Western blot analysis. Overexpression of miR-125b led to a significant reduction of most basal or GnRH-induced selected Gαq/11-mediated pathway factor protein levels. p38 was not significantly affected (n = 7). (**c**) Cells were electroporated with anti-miR-125b or scrambled LNA. Blocking miR-125b increased most tested miR-125b potential targets with the exception of MAP2K7 which was significantly decreased (n = 7). (**d,e**) Effect of altering miR-125b on CREB phosphorylation status. LβT2 cells were electroporated either with anti-miR125b or scramble LNA, or with miR-125b or empty expression vector and then treated with 10 nM GnRHa for 30 min. Extracted proteins were analysed by western blotting using anti-CREB and anti-pCREB, successively and a representative blot is given below the analysis. d; Blocking miR-125b had no effect on the phosphorylation status of CREB. (**e**) Overexpression of miR-125b had no effect on the GnRH-induced phosphorylation of CREB (n ≥ 4). Representative western blot images are given on Fig. S2. *P < 0.05; **P < 0.01; ***P < 0.001.

We first looked for the presence of miR-125b and its potential target mRNAs in RISC. Each complex contains a given targeted mRNA together with its targeting miRNA^26^. The complexes were immunoprecipitated by using a pan-AGO antibody. In addition to miR-125b, mRNAs for *Gna11* (which encodes Gαq/11), *Map2k7, Elk1, Jun* and *Camk2a* were shown to be present in the immunoprecipitated complexes (Fig. 2a) in control cells. All co-immunoprecipitated mRNAs exhibited reduced levels when cells were treated with GnRHa, together with the reduction of miR-125b. In order to confirm a role of miR-125b in the GnRH-induced captures into RISC, we looked for the effects of both overexpression and blocking of miR-125b on signalling effectors content. We confirmed that miR-125b overexpression decreased the protein level of MAP2K7, ELK1 and Gαq/11 (Fig. 2b; see Fig. S2a for representative blots). JUN level was too low in un-stimulated cells but overexpression of miR-125 prevented its GnRH-induced rise (Fig. 2b). Blocking miR-125b increased protein levels of Gαq/11, p38, ELK1, and GnRH-induced JUN (Fig. 2c and Fig. S2b). Unlike most miRNAs, miR-125b usually provokes a down-regulation of its target messengers, by inducing their rapid deadenylation^22^. We showed that overexpression of miR-125b induced a decrease in the basal level of *Map2k7, Elk1, Gna11* and *Mapk14* mRNAs and a marked decline in GnRH-induced *Jun* and *Camk2a* mRNAs (Fig. S1d), in agreement with the variations observed in their cellular encoded protein levels. In addition, *Itrp1* and *Cacna1c* mRNAs were also reduced (Fig. S1d) suggesting that their encoded proteins would also be down-regulated. Conversely, blocking miR-125b enhanced the cellular content in *Map2k7, Mapk14, Itrp1, Cacna1c, Gna11* and *Jun* mRNAs (Fig. S1e). If *Elk1* (and *Camk2a*) mRNA level did not appear significantly increased when miR-125b action was prevented, the level of the encoded ELK1 protein was actually increased (Fig. 2c). Conversely, MAP2K7 level was not increased (rather the reverse) when miR-125b action was prevented despite the rise of its encoding mRNA, suggesting that other factors affecting MAP2K7 turnover may be altered by the treatment. A discrepancy between mRNA (*Mapk14)* and protein levels of p38 was also observed when miR-125b was overexpressed.

*In silico* analysis indicates that CREB is not a potential target of miR-125b. It has been shown that GnRH treatment does not alter the level of CREB but stimulates its PKA-mediated phosphorylation^12^,^27^. Neither overexpression (Fig. 2d and Fig. S2c) nor blocking (Fig. 2e and Fig. S2d) miR-125b had any effect on the GnRH-induced level and phosphorylation state of CREB after 30 min of treatments showing that the coupling to Gαs is still able to convey a GnRH stimulatory signal through the cAMP pathway.

Altogether, these results show that miR-125b targets into RISC mRNAs of a number of GnRH signalling effectors associated with coupling to the Gαq/11-calcium-activated pathway, likely exerting a silencing effect on most GnRH signalling mediators. The absence of effect on the Gαs-cAMP activated pathway keeps an entry door open for GnRH. GnRH treatment leads to a decrease in miR-125b level, allowing full activation of its Gαq/11-dependent signalling and allowing enhanced gonadotropin subunits expression.

### miR-125b is repressed by an NSun2-mediated methylation in gonadotrope cells

NOP2/Sun RNA methyltransferase (NSun2) or MISU for myc-induced SUN-domain-containing protein^28^ was first characterized as responsible for transferring a methyl group from S-adenosyl-L-methionine to cytosine residues (m5C) of transfer RNAs^29^. However, NSun2 has recently been shown to be involved in the repression of miR-125b actions in Hela cells by methylating adenosine into *N*6-methyladenosine (m6A) on specific positions of primary and pre-miR-125b transcripts, thereby inhibiting the processing^30^. NSun2 was also shown to be responsible for the methylation of the mature form of miR-125b, which attenuates the recruitment of miR-125b into RISC^30^.

**Figure 3:**
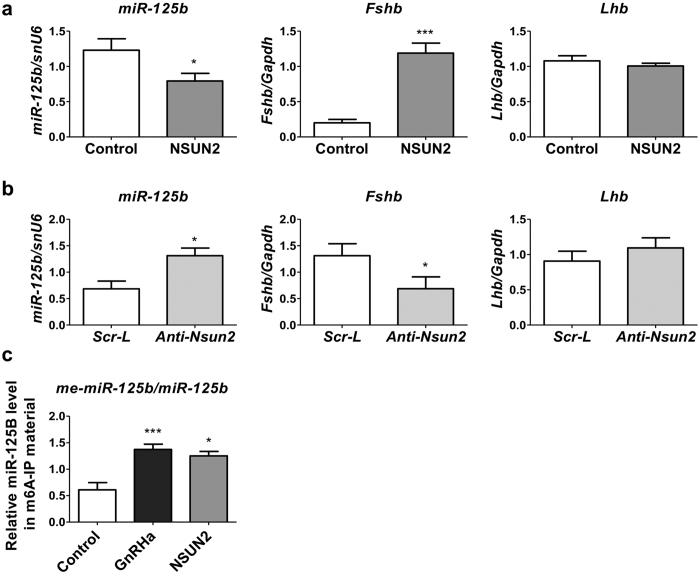
miR-125b is repressed by an NSun2-mediated methylation in gonadotrope cells. (**a,b**) Effect of overexpressing or blocking NSun2. LβT2 cells were electroporated with NSun2 or control expressing vector and miR-125b, *Fshb* and *Lhb* mRNA levels were quantified by qRT-PCR and normalized to snU6 (miR-125b) or to *Gapdh*. (**a**) Overexpression of NSun2 reduced mi-125b and increased *Fshb* mRNA level. *Lhb* mRNA level was not affected (n = 9). (**b**) Blocking NSun2 using an anti-*NSun2* LNA led to inverse effects compared to cells electroporated with a scramble LNA (n = 9). (**c**) Effect of a GnRHa treatment and of overexpressing NSun2 on the methylation status of miR-125b. LβT2 cells were either treated by GnRH (10 nM for 4 h) or electroporated with an NSun2 expressing vector and harvested. Total RNA extract was immunoprecipitated using an anti-m6A antibody and miR-125b was quantified in the immunoprecipitated fraction and normalized to input miR-125b level. Overexpression of NSun2 had a similar stimulatory effect as the GnRHa treatment on the methylation status of miR-125b (n ≥ 4). *P < 0.05; **P < 0.01; ***P < 0.001.

Overexpression of *Nsun2* in LβT2 cells, which reached 2.5-fold its level in control cells (Figure S3a), led to a decrease in miR-125b level down to 60% of its steady-state level (Fig. 3a) and induced an increase in *Fshb* expression (Fig. 3a) and in all identified miR-125b mRNA targets (Fig. S3c). Conversely, the silencing of Nsun2 down to 30% of its cellular content using an anti-Nsun2 LNA (Fig. S3b) induced a doubling in miR-125b level (Fig. 3b), the repression of *Fshb* subunit expression (Fig. 3b) and a decline in most GnRH signalling miR-125b target effectors (Figure S3d). Unlike when altering directly miR-125b levels, either overexpression or blocking NSun2 had no significant effect on *Lhb* endogenous mRNA (Fig. 3a,b). Overexpression of NSun2 had a lower effect on miR-125b level as compared to when blocked using a LNA (down to 60% compared to 10%). *Lhb* was suggested to be more sensitive to the Gαs-mediated stimulation than *Fshb* (see refs 3, 4, 5 for review) so that moderate alteration of Gαq/11 alone might not be sufficient to significantly affect *Lhb* mRNA level. In addition, overexpression of Nsun2 would not only lead to a decrease in miR-125b but may also have a large panel of effects as this methyltransferase is notably known to also methylate messenger as well as transfer RNAs^29^. These additional effects might interfere with those due to the decrease of miR-125b.

To assess a possible role for the methylation of miR-125b in the regulation of gonadotropin subunits expression by GnRH, RNA isolated from LβT2 cells was immunoprecipitated using a highly specific anti-m6A antibody. The methylated fraction of miR-125b increased in the immunoprecipitated fraction in response to GnRHa treatment as well as in cells in which Nsun2 was overexpressed (Fig. 3c).

### GnRH activates NSun2 by a PKA-dependent phosphorylation

**Figure 4:**
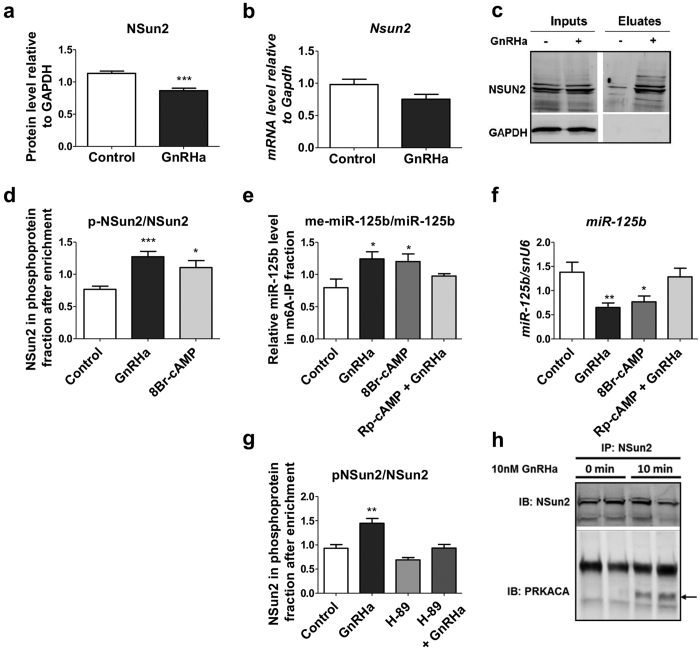
GnRH activates NSun2 by a PKA-dependent phosphorylation. (**a,b**) LβT2 cells were treated by GnRH (10 nM for 4 h). (**a**) Nsun2, quantified by western blot analysis, was down-regulated by the GnRHa treatment (n = 10). (**b**) *Nsun2* mRNA quantified by qRT-PCR was lower albeit non-significantly in GnRH-treated cells relative to control cells (n = 9). (**c,d**) Protein extracts were eluted from a phosphoprotein enrichment column and analysed by western blotting using an anti-NSun2 antibody. (**c**) Representative blot comparing input proteins from GnRH-treated or control cells with eluted proteins from enrichment columns. (**d**) LβT2 cells were either treated by GnRHa (10 nM for 4 h) or the cAMP analogue 8Br-cAMP or were pre-treated with the PKA inhibitor Rp-cAMP before the same GnRHa treatment. Activation of PKA using a 8Br-cAMP treatment had similar increasing effect as GnRHa on the phosphorylation state of NSun2 (n = 4). (**e**) The same treatment had similar increasing effect as GnRHa on the methylated fraction of miR-125b, whereas pre-treatment with the PKA inhibitor Rp-cAMP prevented this effect (n ≥ 4). (**f**) Same treatments had corresponding inverse effects on miR-125b cellular content (n ≥ 8). (**g**) LβT2 cells were either pre-treated with the PKA inhibitor H89 followed or not by a GnRHa treatment (10 nM for 4 h) or treated by GnRHa alone. The basal level of phosphorylation was not affected by the PKA inhibitor treatment alone. The GnRH-induced phosphorylation of NSun2 was prevented by the PKA inhibitor (n ≥ 5). (**h**) LβT2 cells were treated by GnRHa (10 nM for 10 min). Protein extracts were immunoprecipitated using anti-NSun2 antibody. Co-immunoprecipitated proteins were analysed by western blotting using anti-PRKACA. Representative blot comparing immunoprecipitated NSun2 proteins from GnRH-treated or control cells. The catalytic subunit of PKA was associated with NSun2 10 min after the GnRHa-treatment. Representative western blot images are given on Fig. S2. *P < 0.05; **P < 0.01; ***P < 0.001.

Both Nsun2 protein (Fig. 4a and Fig. S2e) and mRNA levels (Fig. 4b) were reduced 4 h after a GnRHa treatment indicating that, in our gonadotrope cell model, the modification of the NSun2 level could hardly be accounted for its activation in response to GnRH. The Aurora-B kinase has been shown to phosphorylate Nsun2 on Ser139^31^. Using a phosphoprotein enrichment method, we observed that the level of NSun2 increased in the phosphoprotein fraction after a GnRHa treatment of LβT2 cells (Fig. 4c,d and Fig. S2f), demonstrating that GnRH induces phosphorylation of NSun2.

As CREB phosphorylation was not affected by modulating miR-125b in LβT2 cells, we investigated the potential role of the cAMP-dependent pathway in the GnRH-induced miR-125b methylation. We observed that increasing intracellular cAMP level using 8Br-cAMP significantly amplified the phosphorylated fraction of NSun2 (Fig. 4d and Fig. S2f) as well as the methylated form of miR-125b (Fig. 4e and Fig. S2g) and reduced miR-125b cellular content (Fig. 4f). Conversely, inhibiting PKA pathway by the inhibitor (H89) did not significantly affect the level of phosphorylation of NSun2 in control cells (Fig. 4g), showing that the basal phosphorylation state of NSun2 is not mediated by PKA. However, the presence of inhibitor prevented the GnRH-induced rise in the phosphorylation state of NSun2 (Fig. 4g). In agreement, pre-treatment with the PKA inhibitor Rp-cAMP suppressed the GnRH-induced miR-125b increase in the methylated fraction (Fig. 4e) and its cellular decrease (Fig. 4f). PKA was shown to be activated within 5–10 min of a GnRH treatment of LβT2 cells^12^. Co-immunoprecipitation experiments using an anti-Nsun2 antibody showed that the catalytic subunit of PKA (PRKACA) was associated with Nsun2, 10 min after a GnRHa treatment (Fig. 4h), further confirming that PKA was directly responsible for the GnRH-induced phosphorylation of NSun2.

### miR-125b/miR-132 opposite regulation in gonadotrope cells

**Figure 5:**
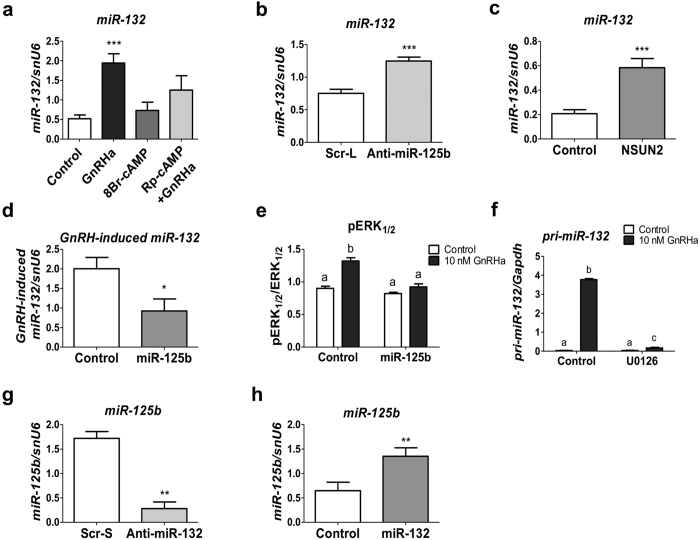
miR-125b/miR-132 opposite regulation in gonadotrope cells. (**a**) LβT2 cells were either treated by GnRHa (10 nM for 4 h) or the cAMP analogue 8Br-cAMP or were pre-treated with the PKA inhibitor Rp-cAMP before the same GnRHa treatment. Inhibition of PKA prevented the GnRH induction of miR-132 (n = 8) (**b–d**) Effect of alteration of miR-125b on miR-132 level. (**b**) LβT2 cells were electroporated with anti-miR-125b or scrambled LNA. Blocking miR-125b increased miR-132 level (n = 8). (**c**) Cells were electroporated with NSun2 or empty expressing vector. Overexpression of NSun2 led to a significant increase of miR-132 expression (n = 9). (**d**) Cells were electroporated with miR-125b or empty expression vector before a GnRHa treatment (10 nM for 4 h). Overexpression of miR-125b led to a significant reduction of the GnRHa-induced miR-132 expression (n = 4). (**e**) Effect of alteration of miR-125b on ERK1/2 phosphorylation status. LβT2 cells were electroporated with miR-125b or empty expression vector and then treated with 10 nM GnRHa for 2 h. Extracted proteins were analysed by western blotting using anti-ERK1/2 and anti-pERK1/2, successively. A representative blot is given on Fig. S2h). The antiserum recognizes both ERK1 (upper band) and ERK2 (lower band). The two signals were summed for quantification. Overexpression of miR-125b prevented the GnRH-induced phosphorylation of ERK1/2 (n = 3). (**f**) LβT2 cells were either treated by GnRHa (10 nM for 8 h) or were pre-treated with the MAPK kinase (MEK)1/2 (U0126) inhibitors before the same GnRHa treatment. Inhibition of ERK1/2 phosphorylation prevented the GnRH-induced expression of AK006051 mRNA encoding miR-132 and miR-212 (n = 3). (**g,h**) Effect of alteration of miR-132 on the miR-125b level in LβT2 cells. (**g**) Cells were electroporated with anti-miR-132 or scrambled LNA. Blocking miR-132 led to a significant decrease in miR-125b level (n = 6). (**h**) Cells were electroporated with a miR-132 expressing vector or an empty vector. Overexpression of miR-132 led to a significant increase of miR-125b expression (n = 5). *P < 0.05; **P < 0.01; ***P < 0.001.

In a previous study, we demonstrated that a rise of miR-132 in GnRH-treated gonadotrope cells was necessary for the stimulation of *Fshb* expression^20^. It was therefore interesting to determine whether the elevation of miR-132 in GnRH-treated gonadotrope cells was induced, or allowed, by the down regulation of miR-125b. Increasing cAMP effect alone was inefficient in enhancing mature miR-132 level (Fig. 5a). However, the GnRH-induced rise in miR-132 was prevented when cells were pre-treated with the PKA inhibitor Rp-cAMP (Fig. 5a), attesting an indispensable role of this pathway in the activation of miR-132 by GnRH. The methylation status of miR-125b is altered in response to PKA activation, potentially allowing an involvement of miR-125b in the regulation of miR-132. Lowering miR-125b level by either using anti-miR-125b LNA or overexpressing NSun2 increased miR-132 level (Fig. 5b,c). Conversely, overexpression of miR-125b prevented the GnRH-induced rise of miR-132 level (Fig. 5d), indicating that miR-125b represses miR-132 expression. In cortical neurons, miR-132 expression is stimulated by the activation of ERK signalling^32^. We show here that overexpression of miR-125b prevented the GnRH-induced phosphorylation of ERK1/2 (Fig. 5e and Fig. S2h). In addition, blocking ERK1/2 phosphorylation by treatment with U0126, a MEK1/2 inhibitor prevented the GnRH-induced rise in the expression of *AK005051* transcript encoding pri-miR-132/212^32^ (Fig. 5f). This result indicates that the GnRH-induced down-regulation of miR-125b would allow the Gαq/11-mediated MEK1/2-induced activation of ERK1/2 and its stimulatory action on miR-132/212 gene transcription.

Conversely, blocking miR-132 induced a decrease in miR-125b level (Fig. 5g) whereas miR-132 overexpression increased miR-125b cellular content (Fig. 5h). Taken together these results demonstrate the existence of a regulatory loop between miR-125b and miR-132, two miRNAs that have opposing effects on gonadotropins expression. In this loop, the GnRH-induced decrease in miR-125b would lead to an up-regulation of miR-132 expression which in turn has a stimulatory effect on miR-125b expression. Such a loop should contribute to a return to steady-state levels of these miRNAs latterly after GnRH stimulation.

### Dynamics of the miR-125b/miR-132 regulatory loop

**Figure 6:**
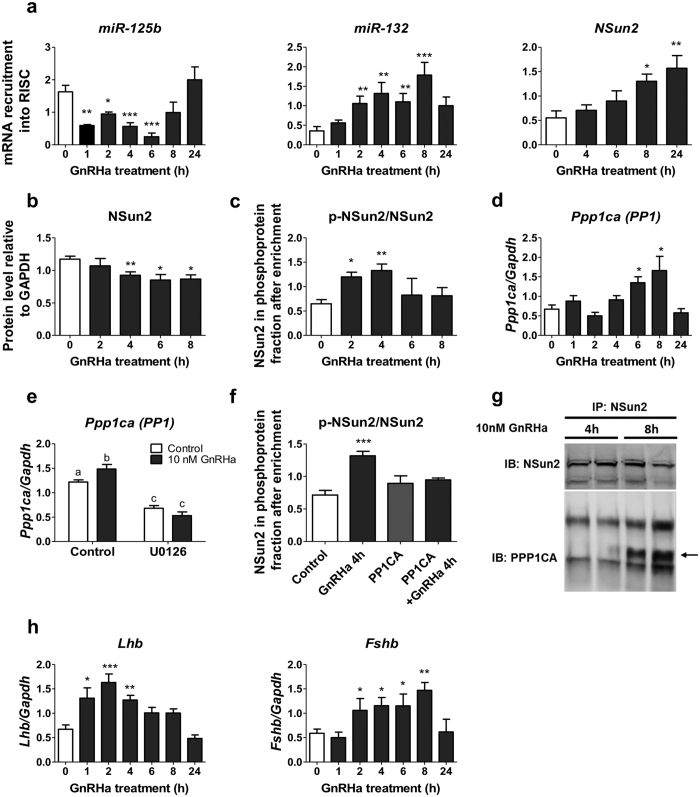
miR-125b/miR-132 regulatory loop involvement in gonadotrope cell desensitization to GnRH. (**a–d**) Dynamics of the GnRH-induced miR-125b/miR-132 regulatory loop. LβT2 cells were treated with 10 nM GnRHa. (**a**) Effect on the recruitment into RISC. miR-125b, miR-132 and *NSun2* mRNA levels were determined on pan-AGO-immunoprecipitated samples and normalized to input level. Recruitment of miR-125b was quickly reduced and kept low until 8 h of treatment. Recruitment of miR-132 was delayed until 2 h and maintained for up to 8 h. *NSun2* mRNA was recruited only after 8 h (n ≥ 5). (**b**) GnRHa treatment induces a decrease in NSun2 protein level (n ≥ 4). (**c**) Protein extracts were eluted from a phosphoprotein enrichment column and analysed by western blotting using an anti-NSun2 antibody. The GnRHa-treatment rapidly induced a strong increase of NSun2 in the phosphoprotein fraction, peaking at 4 h (n ≥ 4). (**d–f**) NSun2 is inactivated by a PP1α-dependent dephosphorylation. d; LβT2 cells were treated with 10 nM GnRHa. *Ppp1ca* mRNA was significantly induced after 6 h of treatment (n ≥ 5). (**e**) LβT2 cells were either pre-treated with the MEK1/2 inhibitors (U0126) or not before treatment with GnRHa (10 nM for 8 h). Inhibition of ERK1/2 phosphorylation prevented the GnRH-induced expression of *Ppp1ca* mRNA (n = 3). (**f**) Cells were electroporated with PP1CA or empty expression vector followed by a GnRHa treatment (10 nM for 4 h). PP1CA overexpression alone had no effect on basal phosphorylation level but prevented the GnRH-induced phosphorylation of NSun2 (n ≥ 3). (**g**) Cells were treated by GnRHa (10 nM for 4 or 8 h). Protein extracts were immunoprecipitated using anti-NSun2 antibody. Co-immunoprecipitated proteins were analysed by western blotting using anti-PP1CA. Representative blot of immunoprecipitated NSun2 proteins from GnRH-treated compared to control cells. The catalytic subunit of PP1α was associated with NSun2 after 8 h of GnRHa-treatment but not after 4 h. (**h**) Cells were treated with 10 nM GnRHa. *Lhb* and *Fshb* mRNAs were significantly induced in GnRH-treated cells relative to control cells and returned to control values after 24 h (n ≥ 5). Representative western blot images are given on Fig. S2M. *P < 0.05; **P < 0.01; ***P < 0.001.

To get a better insight into the temporal sequence of the GnRH response, we performed kinetics experiments. RISC content of miR-125b appeared to rapidly decrease in response to GnRHa stimulation (Fig. 6a). As expected, miR-132 recruitment quickly followed, likely resulting from enhanced gene expression (Fig. 6a). After eight hours of treatment, when the RISC content of miR-132 was at its highest level, miR-125b started to be re-captured and the level of *NSun2* mRNA into RISC started to rise (Fig. 6a) while the cellular content of NSun2 protein decreased (Fig. 6b and Fig. 2i). Interestingly, *in silico* analysis indicated that NSun2 is a potential target of miR-132. In agreement, when miR-132 was overexpressed, *NSun2* mRNA level increased in AGO-immunoprecipitated fraction (Fig. S4a) and the cellular content of Nsun2 protein was lowered (Fig. S4b) while its messenger RNA was unaffected (Fig. S4c). These results confirm that the rise of miR-132 contributes to the return of miR-125b to its steady-state level through an inhibition of NSun2 expression.

As Nsun2 protein level does not necessarily reflect its potential activity, we looked at the phosphorylation status of NSun2 throughout the GnRHa treatment. The NSun2 phosphorylation level was already increased 2 h after GnRH exposure (Fig. 6c and Fig. S2j) in agreement with the observed negative effect on the recruitment of miR-125b into RISC and on its cellular content. After 6 h of treatment, the phosphorylated fraction of Nsun2 was lowered and miR-125b started to rise and be recruited again into RISC.

### NSun2 is inactivated by a PP1α-dependent dephosphorylation

The decrease in the phosphorylation status of NSun2 suggests that it is submitted to the action of a phosphatase. We quantified the mRNA level of a number of phosphatases by qRT-PCR among which the catalytic subunit of PP2, PP2CA (Figure S4d) but only the messenger for the catalytic subunit of PP1α, PPP1CA, exhibited a profile in agreement with the level of phosphorylation of NSun2. *Ppp1ca* increased significantly after 6 h of GnRH treatment (Fig. 6d) *i.e.* just before the decrease of the phosphorylated form of NSun2. Blocking MEK1/2 by using U0126 decreases basal level of *Ppp1ca* and, as for miR-132 primary transcript expression, prevented its GnRH-induced rise (Fig. 6e). This shows that the rise of both *Ppp1ca* and miR-132 relies on the activation of ERK1/2 which is allowed by the lifting of the miR-125b-mediated inhibition of the Gαq/11 pathway. Overexpression of PPP1CA countered the GnRH-induced phosphorylation of NSun2 after 4 h of treatment without affecting the basal level of its phosphorylation (Fig. 6f and Fig. S2k). Co-immunoprecipitation experiment using an anti-NSun2 antibody showed that PPP1CA was associated with NSun2 after 8 h of GnRH treatment (Fig. 6g). These results strongly suggest that the activation of PP1α is responsible for the inactivation of NSun2, the return of miR-125b to its steady-state level and the silencing of the Gαq/11 signalling pathway. This silencing effect is attested by a decrease in *Lhb* and *Fshb* mRNA levels (Fig. 6h).

## Discussion

Although mammalian GnRHR is resistant to homologous desensitization, gonadotropin secretion is significantly reduced or abolished by sustained treatment with GnRH agonists. In the rhesus monkey with radio-frequency induced lesions of the medial basal hypothalamus which ablates most GnRH-secreting neurons, gonadotropin secretion are restored when GnRH is administered intermittently but not when infused continuously^33^. Similarly, *in vivo* treatment with long-lasting GnRH analogues, reduces levels of *Lhb* and *Fshb* transcripts in rat pituitary gland^34^ and the secretion of gonadotropin hormones^35^. More interestingly, in ewes in which GnRH levels are monitored in the pituitary portal blood during the course of the follicular phase, LH secretion surge appears to end several hours before the amplitude and frequency of the GnRH pulses decline, indicating that desensitization also occurs in physiological conditions^36^.

Desensitization of gonadotrope cells to sustained GnRH stimulation has first been attributed to a lack of calcium response^37^ possibly through decreased expression of the IP3 receptor^38^. Expression of several signalling factors was shown to be down-regulated upon prolonged GnRHR stimulation. This is notably the case for Gαq/11^39^, PLCβ1^11^ and several isoforms of PKC^11^. Overexpression of a constitutively active mutant of Gαq/11 induces resistance to GnRH^3^,^11^ further indicating that desensitization to GnRH involves a Gαq/11-mediated pathway.

It was shown that treatment with GnRH of murine LβT2 gonadotrope-derived cells^18^,^19^ or porcine pituitary cells^40^ induces considerable changes in the microtranscriptome. In this study, we show that miR-125b content in RISC dropped within 1 h and was maintained low for 6 h of sustained GnRH exposure and then progressively re-increased to reach its steady-state level after 24 h. This indicated that the repressive effects of miR-125b on targets are transiently lifted for a limited time after the beginning of the GnRH treatment. miR-125b was demonstrated to target several components of the Gαq/11-mediated pathway. If MAP2K7^22^,^23^, p38^23^ and JUN^24^ were already described as effective targets of miR-125b, others, like, Gαq/11, ITPR1, CamK2a or ELK1 were here revealed for the first time to our knowledge. Blocking miR-125b induced an increase in these targets and a stimulation of LH and FSH expression, indicating that the steady-state level of miR-125b is high enough in unstimulated cells to convey its silencing effect on the Gαq/11-mediated pathway. In contrast, the Gαs-mediated pathway remained fully open to stimulation even when miR-125b was overexpressed, in agreement with previous reports showing that only the Gαq/11-mediated pathway is submitted to desensitization^12^. By targeting Gαq/11 and several downstream components of the Gαq/11 activated pathway, the effects of miR-125b are likely to account for most if not all the previously described alterations provoked by sustained exposure to GnRH. Importantly, owing to its silencing effects on Gαq/11-mediated signalisation, miR-125b may be involved in the regulation of a number of G protein-coupled receptors that activate this pathway.

The desensitization effect is due to a return of miR-125b to its steady-state, silencing level, a few hours after the beginning of a continuous exposure to GnRH. The decrease of miR-125b was generated by its methylation induced by the PKA-activated Nsun2 and its return to steady-state level was allowed by a PP1α-provoked dephosphorylation of NSun2, together with a miR-132-mediated decrease of NSun2. In this work, we showed that overexpression of PP1CA prevented the GnRH-induced phosphorylation of NSun2 but did not lower the phosphorylation state of NSun2 in unstimulated cells. Aurora B was shown to phosphorylate human NSun2 at Ser139 and this phosphorylation had an inactivating effect on the methylation on cytosine residues of single-stranded DNA or tRNA^31^. Among the other sites that could potentially be phosphorylated, we identified Ser232 and Ser641 (in mouse) that are located in environments (KRLSS and RKLSS, respectively) conserved among mammalian NSun2 sequences and corresponding to the consensus site for PKA-mediated phosphorylation. Whether the PP1α-resistant phosphorylation sites include Ser139 or not was not determined but our results show that PKA is able to phosphorylate NSun2 on different, PP1α-sensitive sites, possibly Ser232 and/or Ser641. This phosphorylation appears to confer the methyltransferase activity on adenosine to NSun2. Nevertheless, one cannot exclude that the phosphorylation of NSun2 may induce a conformational change that allows interaction with another enzyme that would bear the methyltransferase activity attributed to NSun2.

If miR-132 up-regulation and miR-125b down-regulation are necessary for GnRH to enhance gonadotropins expression, these regulations are expected to occur also when gonadotrope cells are submitted to a pulsatile mode of stimulation. It is likely that new steady-state levels are reached with miR-125b low enough for the Gαq/11-mediated pathway to exert its transcriptional stimulation, allowing miR-132 to be up-regulated. miR-125b down-regulation is dependent on NSun2 activation by a PKA-mediated phosphorylation. Tsutsumi and coll demonstrated^12^ that multiple pulses of GnRH caused multiple pulses of cAMP and PKA activation without desensitization. Such intermittent stimulation of PKA may activate NSun2 intermittently, allowing a fine-tuning in the level of miR-125b. This hypothesis will have to be investigated using perifused gonadotrope cells submitted to a GnRH pulsatile challenge.

The desensitization effect was shown in this study to rely on a regulatory loop between miR-125b and miR-132 that tends to restore steady-state levels of miR-125b and miR-132. This regulatory loop depends on a PKA-mediated activation of NSun2 and a subsequent deactivation induced by PP1α. Since PKA, NSun2 and PP1 are considered ubiquitous, such a regulatory loop should be activated in a number of tissues. miR-125b and miR-132 have opposite effects on dendritic spine morphology and synaptic physiology in hippocampal neurons^21^. It would be interesting to investigate whether the regulatory loop is operating in hippocampal neurones where miR-132 is shown to be down-regulated in Alzheimer’s disease patients^41^. More generally, owing to their opposite effects on synaptic physiology, such a loop might be important during brain development. It is also tempting to speculate that a deregulation of this loop might be responsible for the inverted dynamics between miR-132 and miR-125b (one is up-regulated whereas the other is down-regulated) described in adrenal tissue submitted to an estradiol treatment^42^ or in pituitary adenomas when compared to normal pituitary tissues^43^. Consistent with this hypothesis, the same inverted regulation was observed in human dermal fibroblasts from patients carrying a homozygous loss-of-function mutation in the *Nsun2* gene when compared to fibroblasts heterozygous for the mutation (see Supplemental Table 6 from Hussain *et al*.^44^).

In conclusion, in this paper, we identified a number of new targets for miR-125b showing that miR-125b is able to silence the Gαq/11-mediated signalling of the GnRH response. We also showed that NSun2 is a target of miR-132. We revealed that the NSun2-mediated methylation on m6A is activated by a PKA-mediated phosphorylation and inactivated by a PP1-catalyzed dephosphorylation. These three enzymes participate in a regulatory loop between miR-125b and miR-132 that tends to restore steady-state levels of miR-125b and miR-132. The activation of this regulatory loop is shown to be responsible for the desensitization of gonadotrope cells to GnRH.

## Materials and Methods

### Products, plasmid constructs and antibodies

GnRH agonist (GnRHa), [D-Trp6]-LHRH or triptorelin was purchased from Sigma-Aldrich. PKA activator 8-Br-cAMP and inhibitors Rp-cAMP and H89 were purchased from VWR International. MAPK kinase (MEK)1/2 (U0126) inhibitor was purchased from Sigma. Cell culture reagents were from Life Technologies. Plasmid expressing miR-125b-1 (pmCherry) is from Addgene (#58990). Plasmid expressing miR-132 (pDsRed2-C1) was previously described^20^. The NSUN2 expression vector (human *Nsun2* coding sequence inserted into pCDNA4-Myc His A) was a gift of Dr. M. Frye (Cancer Research, Cambridge, UK). The PP1CA expressing vector (Myc-His tagged PP1CA in pcDNA3-1) was a gift of Dr. L. Neckers (Center for Cancer Research, NCI, Bethesda, MD). The (−2000/+698) rat *Fshb-*Luc reporter pXP2 vector was a gift from Pr. UB Kaiser (Harvard Medical School, Boston, MA). The (−2018/+6) rat *Lhb* promoter sequence was cloned from rat genomic DNA using primers listed in Table S1. The amplified sequence was inserted 5′ to the coding sequence of luciferase into the multiple cloning site of pGL3 vector (Clontech). Anti-PP1CA antibody^45^ was a gift of Dr. A. Nairn (Rockefeller Institute, New-York, NY). Anti-NSun2 (Meth1) was a gift of Dr. M Frye^46^. Anti-PRKACA was a gift from Dr. BA Hemmings^47^ (Friedrich Miescher Institute for Biochemical Research, Basel, Ch). Anti-MAP2K7 (4172S), anti-GAPDH-HRP (D16H11), anti-p38 MAPK (D13E1), anti-pCREB (87G3), anti-CREB (48H2) and anti-ELK1 (9182S) were purchased from Cell Signalling Technology; anti-JUN (H-79) was from Santa Cruz Biotechnology, anti-Gαq/11 (06–709), anti-pan AGO (clone 2A8) and Anti-N6-methyladenosine (m6A, ABE572) from Merck Millipore.

### Animals, cell culture and treatment

Experiments were conducted according to a protocol that was approved by the institutional animal care and use committee of Paris Diderot University (CEEA40).

Wistar rats anterior pituitary glands were dissected from adult (300–450 g) male rats (Janvier, CERJ) and cells were dispersed as previously described^48^. Cells were electroporated (1.2 × 10^6^ cells/tip) with pmCherry-miR-125b or empty vector as described below and seeded in Ham F-10 medium supplemented with 10% fetal bovine serum (FBS). After overnight incubation, the medium was replaced by the same medium complemented with 0.5% penicillin/streptomycin (P/S - Sigma-Aldrich). After 48 h, cells were starved overnight in serum-free medium then treated for 4 h with GnRHa in the starvation medium.

Mouse pituitary gonadotrope LβT2 cells, provided by Pr. P. Mellon (Department of Reproductive Medicine, University of California, San Diego, CA)^49^,^50^, were maintained in monolayer cultures with DMEM (Invitrogen) supplemented with 10% FBS, 0.5% P/S. Cells at passages 13–19 were plated at a density of 0.5 × 10^6^ cells/cm^2^ in triplicates in multi-well plates or 6-cm dishes coated with 30 μg/mL poly-L-lysine (Sigma-Aldrich). For GnRHa stimulation, cells were starved overnight in DMEM containing 0.5% P/S and then incubated for 2 to 24 h in fresh starving medium supplemented with 10 nM GnRHa. Cells were also treated 4 h with 1 mM 8Br-cAMP or pre-treated with 1 mM Rp-cAMP, 10 μM H89 or 10 μM U0126 for 30 min before stimulation with GnRHa.

### Electroporation

pcDNA4-Myc-His-hNSUN, pmCherry-miR-125b and pDsRed-miR-132 miRNA expressing or control (empty) vectors as well as anti-NSUN2 LNA (GATCATAGAAGAGAATCTCA), anti-miR-125b LNA (TCACAAGTTAGGGTCTCAGGGA), anti-miR-132/212 LNA (AGACTGTT) and corresponding controls, scrambled-L (CATGTCATGTGTCACATCTCTT) and scrambled-S (TCATACTA), respectively (underlined sequences are of locked nucleic acids or LNA - Eurogentec), were electroporated into LβT2 or rat primary pituitary cells using the Neon^®^ Transfection System (Invitrogen) as previously described^20^.

### Immunoprecipitation of AGO-associated mRNA and RISC Complex

Experimental conditions for RISC immunoprecipitation (RIP) were previously described^20^. Briefly, cells were cross-linked in 1% formaldehyde solution. Ten μg of anti-pan AGO antibody, 2 mg of cellular protein extract and 50 μl of Dynabeads^®^ Protein G (Invitrogen) were incubated at 4 °C overnight. The immunoprecipitated (IP) beads were then washed 3 times and subjected to DNase I (Promega) treatment at 37 °C for 20 min. The reaction was stopped and the cross-linking reversed by heating at 70 °C for 60 min. Co-immunoprecipitated RNAs as well as “input” RNAs were extracted using TRIzol reagent (Invitrogen) following manufacturer’s instructions and subjected to DNase I digestion as described above.

### Measurement of *in vivo* methylation

One μg of anti-m6A antibody, 20 μg of cellular RNA extract and 20 μl of Dynabeads^®^ Protein G (Invitrogen) were incubated in 200 μl of IPP buffer (150 mM NaCl, 0.1% NP-40, 10 mM Tris-HCl [pH 7.4]) containing 1 U/μl of RNasin at 4 °C for 2 h. The immunoprecipitated (IP) beads were then washed 5 times in IPP buffer. miRNAs isolated from the IP beads were subject to poly-A tailing and reverse transcription followed by real-time quantitative PCR analysis as described below.

### Gonadotropin assays

LH and FSH concentrations were measured in cell media using an ELISA method. Micro-titration plates (High binding, Greiner Bio-one) were coated overnight at 4 °C with 10 ng of purified rat LH (NIH I-10) or 3 days with 15 ng of purified rat FSH (NIH I-6) diluted in carbonate buffer. Rat LH (NIH RP3) or rat-FSH (NIH RP2) used as standards or culture media were incubated overnight with anti-rat LH (NIH S11 at 1:4000) or anti-rat FSH (NIH S11 at 1:8000) at 4 °C. Plates were rinsed with PBS containing 1% BSA and 0.1% Tween 20 for 1 h at RT and washed with PBS-0.1% Tween 20 before addition of standards or samples for competition binding (2 h at 4 °C). After removal of unbound material, phosphatase alkaline-labelled secondary antibody (dilution 1:2000, Thermo Scientific) was added and phosphatase alkaline activity was revealed after 1 h at 4 °C with SigmaFast pNPP reagent. The minimum detectable LH and FSH concentrations were 0.2 and 1 ng/ml, respectively. The inter-assay coefficient of variation were <10%.

### Transcript quantification

Total RNA was collected using TRIzol reagent (Invitrogen) according to the manufacturer’s protocol. Real-time PCR was carried out in duplicates in the LightCycler 480 Instrument (Roche Diagnostics) as previously described^20^ using primers given in Table S1. Expression levels were normalized to *Gapdh* mRNA level.

### Micro RNA quantification

One μg of total RNA was poly-adenylated by incubating with 5 U of poly (A) polymerase (New England Biolabs) at 37 °C for 30 min in a final volume of 10 μl. First-strand cDNA was synthesized from poly(A)-tailed RNA using a poly(T)-tailed universal primer (3′UP, Table S1) and SuperScript II (Invitrogen) according to the manufacturer’s protocol. Amplification was carried out in 5 μl of LightCycler 480 SYBR Green I Master Mix (Roche Diagnostics) using miRNA-specific primers and a primer mix (LUP:SUP/1:5) so as to increase specificity, as indicated in Table S1. MicroRNA expression levels were calculated as for mRNA quantification (see above) and normalized to snU6 RNA level.

### Protein extraction and immunoblotting

LβT2 cells were washed after GnRHa treatment or electroporation with ice-cold 50 mM HEPES pH 7.0 and homogenized in RIPA lysis buffer (20 mM Tris-HCl; pH 7.5, 150 mM NaCl, 1 mM Na_2_EDTA, 1 mM EGTA, 1% NP-40, 1% sodium deoxycholate, 2.5 mM sodium pyrophosphate, 1 mM β-glycerophosphate, 1 mM Na_3_VO_4_ and 1 μg/ml leupeptin) supplemented with protease and phosphatase inhibitors (Roche) and 5 mM nicotinamide (Sigma-Aldrich). Homogenates were cleared at 20,000× g for 30 min at 4 °C. Total proteins from supernatant were quantified using BCA assay (Pierce). Equal amounts of protein (15–20 μg) were separated on a 10% SDS-PAGE. After transfer onto nitrocellulose membrane, anti-NSun2 (1/1000), anti-MAP2K7 (1/1000), anti-p38 MAPK (1/1000), anti-pCREB (1/1000), anti-CREB (1/1000), anti-ELK1 (1/1000), anti-JUN (1/1000), anti-Gq/11a (1/1000), anti-PP1CA (1/1000), anti-PRKACA (1/1000), anti-PP1α (1/2000) and anti-GAPDH HRP (1/1000) antibodies were successively used in TBS containing 0,01% Tween 20 (TBS-T) supplemented with 5% BSA. Membranes were washed three times in TBS-T and then incubated with horseradish peroxidase-conjugated secondary antibody in TBS-T/5% BSA for 60 min at room temperature and then washed three times in TBS-T. Proteins were detected by enhanced chemioluminescence detection system (GE Healthcare). Blots were analysed with a Fuji LAS-4000 imager and quantified using Image J software. Total CREB and GAPDH were used as internal loading control for pCREB and other proteins expression, respectively. Membranes were stripped in stripping buffer (2% SDS, 62.5 mM Tris pH 6.8, 114 mM β-mercaptoethanol) for 30 min at 50 °C between each detection.

### NSun2 co-immunoprecipitation of PKA catalytic subunit

Co-immunoprecipitation of endogenously expressed proteins was performed using LβT2 cells incubated with or without 10 nM GnRHa for 10 min. Cells were harvested in RIPA lysis buffer (see above). Extracts were incubated overnight with 5 μg of anti-Nsun2 antibody in the presence of Protein G magnetic Dynabeads^®^ (Invitrogen) and resulting complexes were washed, denatured and eluted according to the manufacturer’s instruction.

### NSun2 co-immunoprecipitation of PP1α catalytic subunit

Co-immunoprecipitation of endogenously expressed proteins was performed using LβT2 cells incubated with 10 nM GnRHa for 4 h or 8 h. Cells were cross-linked in 1% formaldehyde solution for 10 min. Crosslinking was stopped using glycine pH 7 for 5 min. Cells were then harvested in RIPA lysis buffer (see above). Extracts were incubated overnight with 5 μg of anti-Nsun2 antibody in the presence of Protein G magnetic Dynabeads^®^ (Invitrogen) and resulting complexes were washed, uncross-linked and denatured at 95 °C during 30 min and then eluted in 2x sample buffer (4% SDS, 20% Glycerol, 0.12 M Tris pH 6.8, and 10% β-mercapthoethanol).

### Phosphoprotein enrichment

Following cell lysis, proteins were enriched in a phosphoprotein enrichment column (Thermo Scientific Phosphoprotein Enrichment Kit, Thermo Fisher Scientific), according to the manufacturer’s instructions. Briefly, 3.5 mg of protein from each LβT2 cell lysate was applied to a phosphoprotein column containing a proprietary enrichment gel. The samples were incubated in the column for 30 min at 4 °C and washed. Retained proteins were eluted with five column washes with elution buffer. Phosphoprotein content, typically yielding 15–25% of the total protein loaded, were determined using the BCA assay.

### Luciferase assay

Cells were electroporated using the Neon^®^ Transfection System (Invitrogen) as previously described and plated into 96-well plates (1.5 10^4^ cells/well) in DMEM with 10% FBS. Cells were co-transfected with 0.2 μg of *Fshb*-Luc or *Lhb*-Luc reporter or control vectors, 0.05 μg of Renilla luciferase-reporter (Promega), a reporter plasmid driven by a Herpes virus thymidine kinase (TK) promoter used as a control for transfection efficiency and 0.2 μg of miRNA expressing or control vector as well as 100 nM of LNA-anti-miR or scrambled anti-miR. After overnight incubation, cells were harvested and luciferase activities were measured using the Dual-Luciferase^®^ Reporter Assay System (Promega) as instructed by the manufacturer.

### Statistical analysis

All values are given as mean ± S.E.M. of at least three (the actual number is given by the value “n”) independent experiments. Statistical differences were first determined using one way ANOVA (GraphPad software) followed by Dunnett’s t test for multiple comparisons and Student’s t test for pair-wise comparisons. Statistical significance was defined as P < 0.05.

## Additional Information

**How to cite this article**: Lannes, J. *et al*. A regulatory loop between miR-132 and miR-125b involved in gonadotrope cells desensitization to GnRH. *Sci. Rep.* **6**, 31563; doi: 10.1038/srep31563 (2016).

## Supplementary Material

Supplementary Information

## References

[b1] Seeburg P. H., Mason A. J., Stewart T. A. & Nikolics K. The mammalian GnRH gene and its pivotal role in reproduction. Recent progress in hormone research 43, 69–98 (1987). 3306841 10.1016/b978-0-12-571143-2.50008-3

[b2] Millar R. P. . Gonadotropin-releasing hormone receptors. Endocrine reviews 25, 235–275 (2004). 15082521 10.1210/er.2003-0002

[b3] Liu F. . Involvement of both G(q/11) and G(s) proteins in gonadotropin-releasing hormone receptor-mediated signaling in L beta T2 cells. The Journal of biological chemistry 277, 32099–32108 (2002). 12050161 10.1074/jbc.M203639200 PMC2930616

[b4] Cohen-Tannoudji J., Avet C., Garrel G., Counis R. & Simon V. Decoding high Gonadotropin-releasing hormone pulsatility: a role for GnRH receptor coupling to the cAMP pathway? Frontiers in endocrinology 3, 107 (2012). 22969749 10.3389/fendo.2012.00107 PMC3431540

[b5] Perrett R. M. & McArdle C. A. Molecular mechanisms of gonadotropin-releasing hormone signaling: integrating cyclic nucleotides into the network. Frontiers in endocrinology 4, 180 (2013). 24312080 10.3389/fendo.2013.00180 PMC3834291

[b6] Pawson A. J. . Mammalian type I gonadotropin-releasing hormone receptors undergo slow, constitutive, agonist-independent internalization. Endocrinology 149, 1415–1422 (2008). 18039780 10.1210/en.2007-1159

[b7] Emons G. & Schally A. V. The use of luteinizing hormone releasing hormone agonists and antagonists in gynaecological cancers. Human reproduction (Oxford, England) 9, 1364–1379 (1994). 10.1093/oxfordjournals.humrep.a138714 7962452

[b8] Barbieri R. L. Hormone treatment of endometriosis: the estrogen threshold hypothesis. American journal of obstetrics and gynecology 166, 740–745 (1992). 1536260 10.1016/0002-9378(92)91706-g

[b9] Kiesel L. A., Rody A., Greb R. R. & Szilagyi A. Clinical use of GnRH analogues. Clinical endocrinology 56, 677–687 (2002). 12072036 10.1046/j.1365-2265.2002.01291.x

[b10] Liu F., Austin D. A. & Webster N. J. Gonadotropin-releasing hormone-desensitized LbetaT2 gonadotrope cells are refractory to acute protein kinase C, cyclic AMP, and calcium-dependent signaling. Endocrinology 144, 4354–4365 (2003). 12960037 10.1210/en.2003-0204

[b11] Liu F., Ruiz M. S., Austin D. A. & Webster N. J. Constitutively active Gq impairs gonadotropin-releasing hormone-induced intracellular signaling and luteinizing hormone secretion in LbetaT2 cells. Molecular endocrinology (Baltimore, Md.) 19, 2074–2085 (2005). 10.1210/me.2004-0145 15878957

[b12] Tsutsumi R., Mistry D. & Webster N. J. Signaling responses to pulsatile gonadotropin-releasing hormone in LbetaT2 gonadotrope cells. The Journal of biological chemistry 285, 20262–20272 (2010). 20406815 10.1074/jbc.M110.132662 PMC2888439

[b13] He L. & Hannon G. J. MicroRNAs: small RNAs with a big role in gene regulation. Nature reviews. Genetics 5, 522–531 (2004). 10.1038/nrg1379 15211354

[b14] Bartel D. P. MicroRNAs: genomics, biogenesis, mechanism, and function. Cell 116, 281–297 (2004). 14744438 10.1016/s0092-8674(04)00045-5

[b15] Brodersen P. & Voinnet O. Revisiting the principles of microRNA target recognition and mode of action. Nature reviews. Molecular cell biology 10, 141–148 (2009). 19145236 10.1038/nrm2619

[b16] Wang H. . Gonadotrope-specific deletion of Dicer results in severely suppressed gonadotropins and fertility defects. The Journal of biological chemistry 290, 2699–2714 (2015). 25525274 10.1074/jbc.M114.621565 PMC4317015

[b17] Wang H., Hastings R., Miller W. L. & Kumar T. R. Fshb-iCre mice are efficient and specific Cre deleters for the gonadotrope lineage. Molecular and cellular endocrinology 419, 124–138 (2016). 26472536 10.1016/j.mce.2015.10.006 PMC4684453

[b18] Godoy J., Nishimura M. & Webster N. J. Gonadotropin-releasing hormone induces miR-132 and miR-212 to regulate cellular morphology and migration in immortalized LbetaT2 pituitary gonadotrope cells. Molecular endocrinology (Baltimore, Md.) 25, 810–820 (2011). 10.1210/me.2010-0352 PMC3082323 21372146

[b19] Yuen T., Ruf F., Chu T. & Sealfon S. C. Microtranscriptome regulation by gonadotropin-releasing hormone. Molecular and cellular endocrinology 302, 12–17 (2009). 19356622 10.1016/j.mce.2008.12.013 PMC2683625

[b20] Lannes J. . Rapid communication: A microRNA-132/212 pathway mediates GnRH activation of FSH expression. Molecular endocrinology (Baltimore, Md.) 29, 364–372 (2015). 10.1210/me.2014-1390 PMC5414753 25635942

[b21] Edbauer D. . Regulation of synaptic structure and function by FMRP-associated microRNAs miR-125b and miR-132. Neuron 65, 373–384 (2010). 20159450 10.1016/j.neuron.2010.01.005 PMC5018398

[b22] Wu L., Fan J. & Belasco J. G. MicroRNAs direct rapid deadenylation of mRNA. Proceedings of the National Academy of Sciences of the United States of America 103, 4034–4039 (2006). 16495412 10.1073/pnas.0510928103 PMC1449641

[b23] Zhang L., Ge Y. & Fuchs E. miR-125b can enhance skin tumor initiation and promote malignant progression by repressing differentiation and prolonging cell survival. Genes & development 28, 2532–2546 (2014). 25403182 10.1101/gad.248377.114 PMC4233245

[b24] Kappelmann M., Kuphal S., Meister G., Vardimon L. & Bosserhoff A. K. MicroRNA miR-125b controls melanoma progression by direct regulation of c-Jun protein expression. Oncogene 32, 2984–2991 (2013). 22797068 10.1038/onc.2012.307

[b25] Counis R. . Gonadotropin-releasing hormone and the control of gonadotrope function. Reproduction, nutrition, development 45, 243–254 (2005). 10.1051/rnd:2005017 15982451

[b26] Wang Y. . Structure of an argonaute silencing complex with a seed-containing guide DNA and target RNA duplex. Nature 456, 921–926 (2008). 19092929 10.1038/nature07666 PMC2765400

[b27] Thompson I. R. . GnRH pulse frequency-dependent stimulation of FSHbeta transcription is mediated via activation of PKA and CREB. Molecular endocrinology (Baltimore, Md.) 27, 606–618 (2013). 10.1210/me.2012-1281 PMC3607701 23393127

[b28] Frye M. & Watt F. M. The RNA methyltransferase Misu (NSun2) mediates Myc-induced proliferation and is upregulated in tumors. Curr Biol 16, 971–981 (2006). 16713953 10.1016/j.cub.2006.04.027

[b29] Sibbritt T., Patel H. R. & Preiss T. Mapping and significance of the mRNA methylome. Wiley interdisciplinary reviews. RNA 4, 397–422 (2013). 23681756 10.1002/wrna.1166

[b30] Yuan S. . Methylation by NSun2 represses the levels and function of microRNA 125b. Molecular and cellular biology 34, 3630–3641 (2014). 25047833 10.1128/MCB.00243-14 PMC4187725

[b31] Sakita-Suto S. . Aurora-B regulates RNA methyltransferase NSUN2. Mol Biol Cell 18, 1107–1117 (2007). 17215513 10.1091/mbc.E06-11-1021 PMC1805108

[b32] Remenyi J. . Regulation of the miR-212/132 locus by MSK1 and CREB in response to neurotrophins. The Biochemical journal 428, 281–291 (2010). 20307261 10.1042/BJ20100024

[b33] Belchetz P. E., Plant T. M., Nakai Y., Keogh E. J. & Knobil E. Hypophysial responses to continuous and intermittent delivery of hypopthalamic gonadotropin-releasing hormone. Science (New York, N.Y.) 202, 631–633 (1978). 10.1126/science.100883 100883

[b34] Lerrant Y. . Expression of gonadotropin-releasing hormone (GnRH) receptor gene is altered by GnRH agonist desensitization in a manner similar to that of gonadotropin beta-subunit genes in normal and castrated rat pituitary. Endocrinology 136, 2803–2808 (1995). 7789305 10.1210/endo.136.7.7789305

[b35] Garrel G. . Evidence that gonadotropin-releasing hormone stimulates gene expression and levels of active nitric oxide synthase type I in pituitary gonadotrophs, a process altered by desensitization and, indirectly, by gonadal steroids. Endocrinology 139, 2163–2170 (1998). 9529006 10.1210/endo.139.4.5890

[b36] Moenter S. M., Caraty A., Locatelli A. & Karsch F. J. Pattern of gonadotropin-releasing hormone (GnRH) secretion leading up to ovulation in the ewe: existence of a preovulatory GnRH surge. Endocrinology 129, 1175–1182 (1991). 1874164 10.1210/endo-129-3-1175

[b37] McArdle C. A. . Desensitization of gonadotropin-releasing hormone action in alphaT3-1 cells due to uncoupling of inositol 1,4,5-trisphosphate generation and Ca2^+^ mobilization. The Journal of biological chemistry 271, 23711–23717 (1996). 8798594 10.1074/jbc.271.39.23711

[b38] Willars G. B. . Rapid down-regulation of the type I inositol 1,4,5-trisphosphate receptor and desensitization of gonadotropin-releasing hormone-mediated Ca2^+^ responses in alpha T3-1 gonadotropes. The Journal of biological chemistry 276, 3123–3129 (2001). 11069921 10.1074/jbc.M008916200

[b39] Shah B. H. & Milligan G. The gonadotrophin-releasing hormone receptor of alpha T3-1 pituitary cells regulates cellular levels of both of the phosphoinositidase C-linked G proteins, Gq alpha and G11 alpha, equally. Molecular pharmacology 46, 1–7 (1994). 8058044

[b40] Ye R. S. . Differentially expressed miRNAs after GnRH treatment and their potential roles in FSH regulation in porcine anterior pituitary cell. PloS one 8, e57156 (2013). 23451171 10.1371/journal.pone.0057156 PMC3579806

[b41] Wong H. K. . De-repression of FOXO3a death axis by microRNA-132 and -212 causes neuronal apoptosis in Alzheimer’s disease. Human molecular genetics 22, 3077–3092 (2013). 23585551 10.1093/hmg/ddt164 PMC13387022

[b42] Hu Z. . Hormonal regulation of microRNA expression in steroid producing cells of the ovary, testis and adrenal gland. PloS one 8, e78040 (2013). 24205079 10.1371/journal.pone.0078040 PMC3810252

[b43] Palumbo T. . Functional screen analysis reveals miR-26b and miR-128 as central regulators of pituitary somatomammotrophic tumor growth through activation of the PTEN-AKT pathway. Oncogene 32, 1651–1659 (2013). 22614013 10.1038/onc.2012.190 PMC4034118

[b44] Hussain S. . NSun2-mediated cytosine-5 methylation of vault noncoding RNA determines its processing into regulatory small RNAs. Cell reports 4, 255–261 (2013). 23871666 10.1016/j.celrep.2013.06.029 PMC3730056

[b45] da Cruz e Silva E. F. . Differential expression of protein phosphatase 1 isoforms in mammalian brain. The Journal of neuroscience: the official journal of the Society for Neuroscience 15, 3375–3389 (1995). 7751917 10.1523/JNEUROSCI.15-05-03375.1995 PMC6578208

[b46] Hussain S. . The mouse cytosine-5 RNA methyltransferase NSun2 is a component of the chromatoid body and required for testis differentiation. Molecular and cellular biology 33, 1561–1570 (2013). 23401851 10.1128/MCB.01523-12 PMC3624257

[b47] Garrel G., McArdle C. A., Hemmings B. A. & Counis R. Gonadotropin-releasing hormone and pituitary adenylate cyclase-activating polypeptide affect levels of cyclic adenosine 3′,5′-monophosphate-dependent protein kinase A (PKA) subunits in the clonal gonadotrope alphaT3-1 cells: evidence for cross-talk between PKA and protein kinase C pathways. Endocrinology 138, 2259–2266 (1997). 9165009 10.1210/endo.138.6.5187

[b48] Garrel G. . Sustained gonadotropin-releasing hormone stimulation mobilizes the cAMP/PKA pathway to induce nitric oxide synthase type 1 expression in rat pituitary cells *in vitro* and *in vivo* at proestrus. Biology of reproduction 82, 1170–1179 (2010). 20181617 10.1095/biolreprod.109.082925

[b49] Thomas P., Mellon P. L., Turgeon J. & Waring D. W. The L beta T2 clonal gonadotrope: a model for single cell studies of endocrine cell secretion. Endocrinology 137, 2979–2989 (1996). 8770922 10.1210/endo.137.7.8770922

[b50] Turgeon J. L. Gonadotropin-releasing hormone neuron cell biology. Trends in endocrinology and metabolism: TEM 7, 55–56 (1996). 18406725 10.1016/1043-2760(95)00230-8

