# Corrigendum: A regulatory loop between miR-132 and miR-125b involved in gonadotrope cells desensitization to GnRH

**DOI:** 10.1038/srep34676

**Published:** 2016-10-05

**Authors:** Jérôme Lannes, David L’hôte, Ambra Fernandez-Vega, Ghislaine Garrel, Jean-Noël Laverrière, Joëlle Cohen-Tannoudji, Bruno Quérat

Scientific Reports 6: Article number: 3156310.1038/srep31563; published online: 08 19 2016; updated: 10 05 2016

The original version of this Article contained a typographical error in the spelling of the author Joëlle Cohen-Tannoudji, which was incorrectly given as Joelle-Cohen-Tannoudji.

The Author Contributions statement,

“J.L. and B.Q. conceived and designed the experiments. J.L., D.L., A.F.-V., G.G. and J.-N.L. performed the experiments. J.L., J.-C.-T. and B.Q. analysed the data and wrote the paper. All authors discussed the results and reviewed the manuscript”.

now reads:

“J.L. and B.Q. conceived and designed the experiments. J.L., D.L., A.F.-V., G.G. and J.-N.L. performed the experiments. J.L., J.C.-T. and B.Q. analysed the data and wrote the paper. All authors discussed the results and reviewed the manuscript”.

These errors have now been corrected in the PDF and HTML versions of the Article.

