# Supplementary material for: A regulatory loop between miR-132 and miR-125b involved in gonadotrope cells desensitization to GnRH
Source: Sci Rep. 2016 Aug 19;6:31563. doi: 10.1038/srep31563 (PMC4990909; doi:10.1038/srep31563)
Supplement: Supplementary Information [file srep31563-s1.pdf]

A regulatory loop between miR-132 and miR-125b involved in gonadotrope cells desensitization to GnRH

Jérôme Lannes, David L'hôte, Ambra Fernandez-Vega, Ghislaine Garrel, Jean-Noël Laverrière, Joëlle-Cohen-Tannoudji and Bruno Quérat.

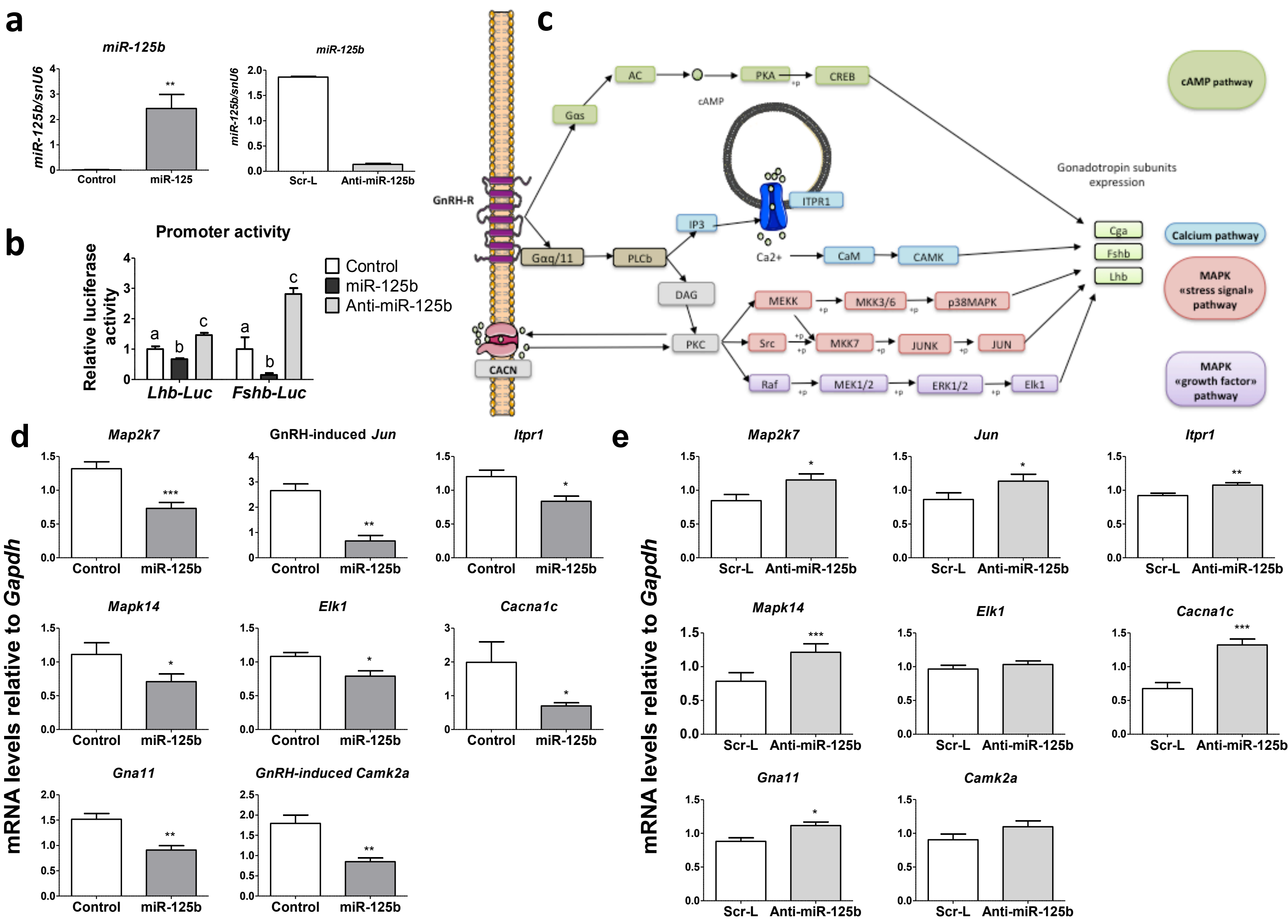

Figure S1: miR-125b overexpression or blocking on miR-125b level (a), LHb and FSHb promoter activity (b), and GnRH signalling effectors (c) expression (d; e) in LβT2 cells

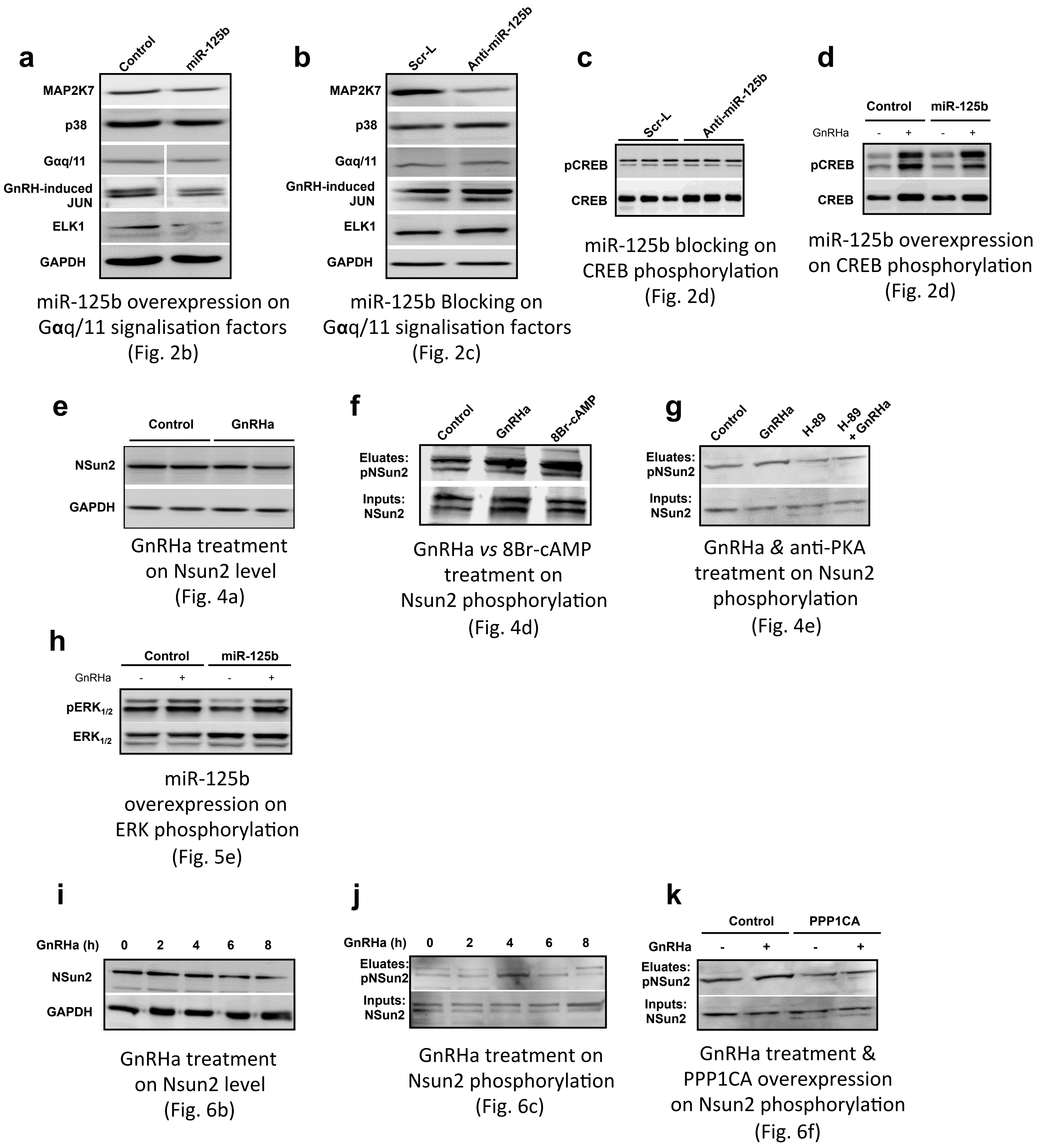

Figure S2: Representative blots

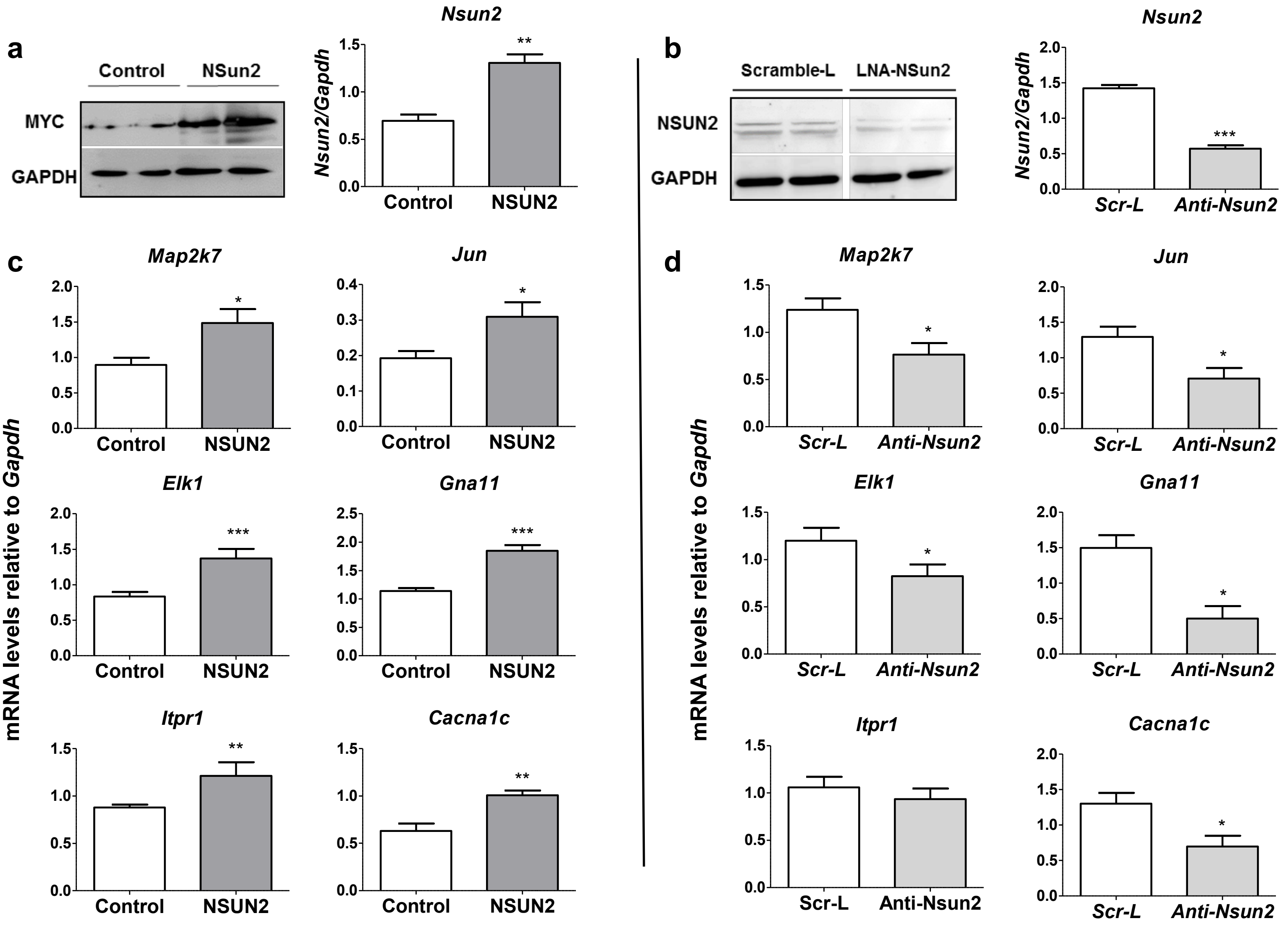

Figure S3: Effects of NSun2 on selected Gαq/11 signalisation factors expression in LβT2 cells

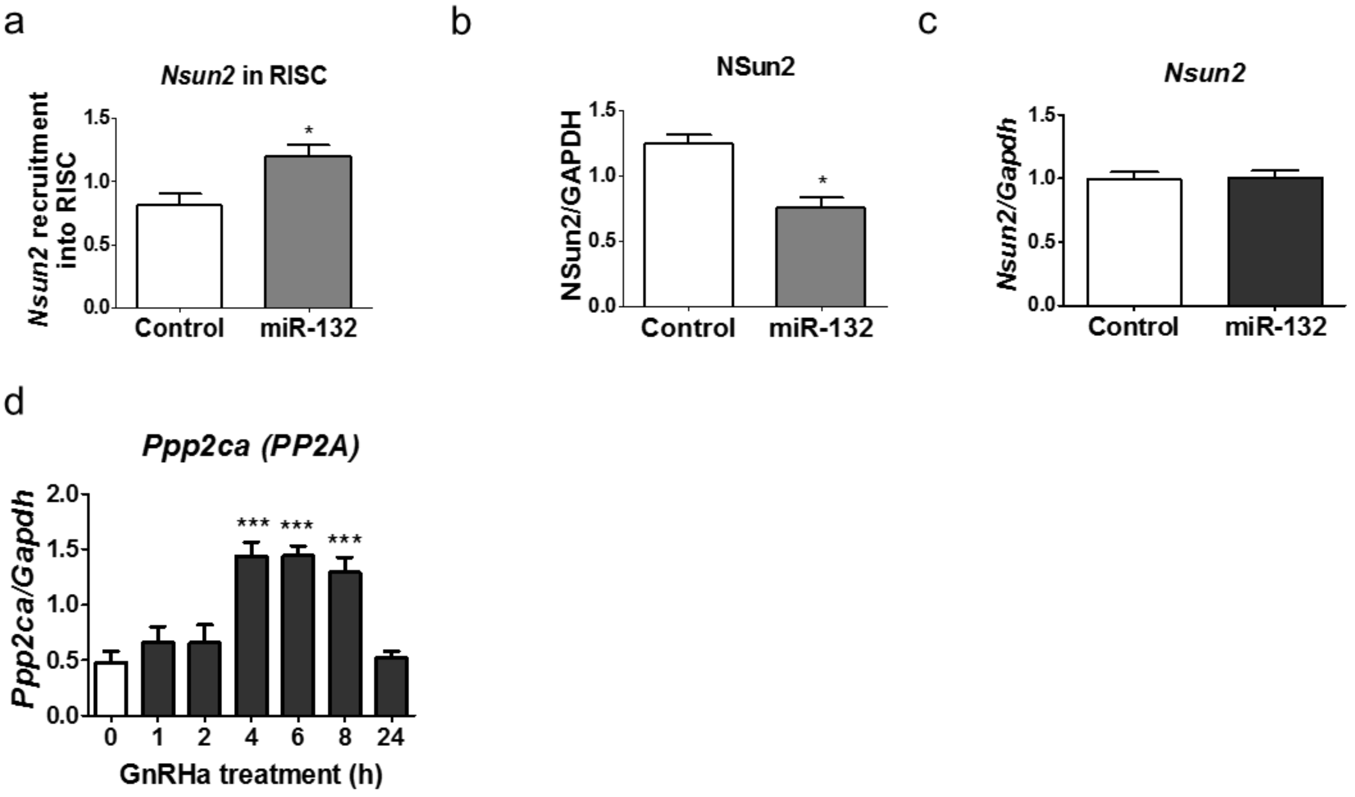

Figure S4: Effects of miR-132 overexpression on NSun2 and PP2CA mRNA expression in LβT2 cells

Table S1. Oligonucleotide primer sequences.

Gene	Forward primer (5'→3')	Reverse primer (3'→5')
**Cloning primer**		
Lhb promoter	GTCTGGGGGTGAGAGAGTTG	GATACCTTCCCTACCTTGGGCA
**Poly(A)tailing**		
3'UP	AAGCAGTGGTATCAACGCAGAGTAC(T)30VN	
**miRNA specific q-PCR primers**		
miR-132	TAACAGTCTACAGCCATGGTCG	
miR-125b	TCCCTGAGACCCTAACTTGTGAA	
snU6	TGGCCCCTGCGCAAGGATG	
LUP		CTAATACGACTCACTATAGGGCAAGCAGTGGTATCAACGCAGAGT
SUP		CTAATACGACTCACTATAGGGC
**mRNA specific primers**		
Gapdh	AGGTCGGTGTGAACGGATTTG	TGTAGACCATGTAGTTGAGGTCA
Fshb	GTGCGGGCTACTGCTACACT	CAGGCAATCTTACGGTCTCG
Lhb	AGCTCGCTGACCACCATC	AATGGTCTGATAGGGTTGAAGC
Nsun2	ACAAACGTCAGCCCAAGGT	GCCACATGGCTGGATACC

Ppp1ca	CAGCCATTGTGGATGAGAAG	CTAATCTGCTCCATGGATTGC
Ppp2ca	ATGGACGAGAAGTTGTTCACC	CAGTGACTGGACATCGAACCT
Map2k7	GCCCAGGCCCATTATTGT	GTTGGCCAGTGGGAGTTG
Jun	CCTGTGCGAACTGGTATGAG	TGCCACCGAGACTGTAAAGA
Elk1	TGCTCCCCACACATACCTTGA	ACTGGACGGAAACTGGAAGGA
Gna11	GCAGAACAAGGCCAATGCA	GCATTCACATACTGGTGCTCAAA
Itpr1	GGCATCTTTGGAGGAAGTGA	ACCCTGAGGAAGGTTCTGC
Cacna1c	GCCCTTCTTGTGCTCTTCGT	GTTGGTGATGCCGTGCTT
Map2k14	AACCAGACAGTGGATATTTGGTC	TCTGCTGAAGCTGGTTAATATGG
Cam2ka	CCTGGCCATAGAGGTGGA	GGGGAGAGGTATCCAGGTGT
